# Conference Report: QUEST FOR EXCELLENCE VI Gaithersburg, MD February 7–9, 1994

**DOI:** 10.6028/jres.099.060

**Published:** 1994

**Authors:** Cheryl Parrot, Robert E. Chapman

**Affiliations:** Office of Quality Programs, National Institute of Standards and Technology, Gaithersburg, MD 20899-0001

## 1. Introduction

As part of the national quality improvement campaign, industry and government have joined together to establish the Malcolm Baldrige National Quality Award. Created by public law in 1987, the Award promotes an understanding of quality excellence, greater awareness of quality as a crucial competitive element, and sharing of information and strategies. There are three categories in which companies may compete: manufacturing, service and small business. Although up to two Awards may be given annually in each of the three categories, and each year many companies apply, to date the maximum number of Baldrige Awards granted in one year is five. In 1989 and 1993, only two companies received the honor. While the criteria against which companies assess their quality Programs evolve, the standards for judging and winning the Award are exceedingly high.

Each year the Quest for Excellence provides quality-seeking businesses the opportunity to listen and question the winning companies, and to learn their strategies for adapting and implementing total quality management (TQM) into their professional lives. In his keynote address, Deputy Secretary of Commerce David J. Barram noted that quality management, as exemplified by the Baldrige Award winners, has a measurable effect, not only on the winners, but on the nation’s economy. The two most recent winners, Eastman Chemical Company and Ames Rubber Corporation, are in the vanguard of companies which have moved to reshape U.S. competitiveness. Barram remarked that the economic effects of quality management have been so dramatic that President Clinton was proposing a 4-million-dollar initiative to extend the scope of the Baldrige Award to the fields of health care and education. Secretary of Commerce Ron Brown and President Clinton had made it clear, Barram said, that this administration, both pro-business and avowed steward of the environment, would, “… urge all U.S. business to follow the models of Eastman and Ames in the combat of the global marketplace … where they take no prisoners.”

The companies that apply for the Malcolm Baldrige National Quality Award each year write extensive descriptions of their business practices, showing the Board of Examiners for the Award how they have implemented the philosophy and techniques of total quality management to meet the stringent criteria of the Baldrige Award. This Conference Report, a summary of the comments by representatives of the winning companies, is organized much as the presentations themselves were. Plenary sessions based on the Baldrige Award criteria are: Leadership (Sec. 2.); Information and Analysis (Sec. 3.); Strategic Quality Planning (Sec. 4.); Human Resource Development and Management (Sec. 5.); Management of Process Quality/Quality and Operatonal Results (Sec. 6.); and Customer Focus and Satisfaction (Sec. 7.). In addition, there were special sessions on Public Responsibility and Corporate Citizenship (Sec. 8.) and on the role of Quality in Research and Development (Sec. 9.). The final section, a Conference Summary, is a synopsis of comments by Dr. Joseph M. Juran, a renowned expert in quality management since 1924. Dr. Juran founded the Juran Institute, dedicated to providing education, training, and consulting in quality to organizations worldwide.

## 2. Leadership

In the experience of Earnest Deavenport, Chairman and Chief Executive Officer of Eastman, and of Joel Marvil, Ames’ President and Chief Executive Officer, success in applying the concept of total quality management is impossible without the strong commitment, involvement, and personal support of an organization’s senior management team.

For Eastman, according to Deavenport, the journey began with a wake-up call in the late 1970s, when his company of over 17,000 employees was losing market share in a major product line, one that it had invented, patented, and licensed others to make. They saved the product line, making a conscious decision to adopt the tools and techniques of quality, and soon learned that integrated processes and systems and the social aspects of quality are critical for a world-class company

Founded in 1949, Ames supplies elastomeric rollers, such as those used in typewriters, to office equipment manufacturers. To meet the demands of a marketplace changing from typewriters to computers, photocopiers, and printers, Ames had a choice of extinction or evolution. Its evolution however, was insufficient to maintain a competitive stance with offshore companies. In the 1980s Ames sought a new culture; the company used more innovative design, more efficient manufacturing techniques, and most importantly, it expanded the involvement of its 450 employees through training programs and built a teamwork philosophy

**Fig. 1:**
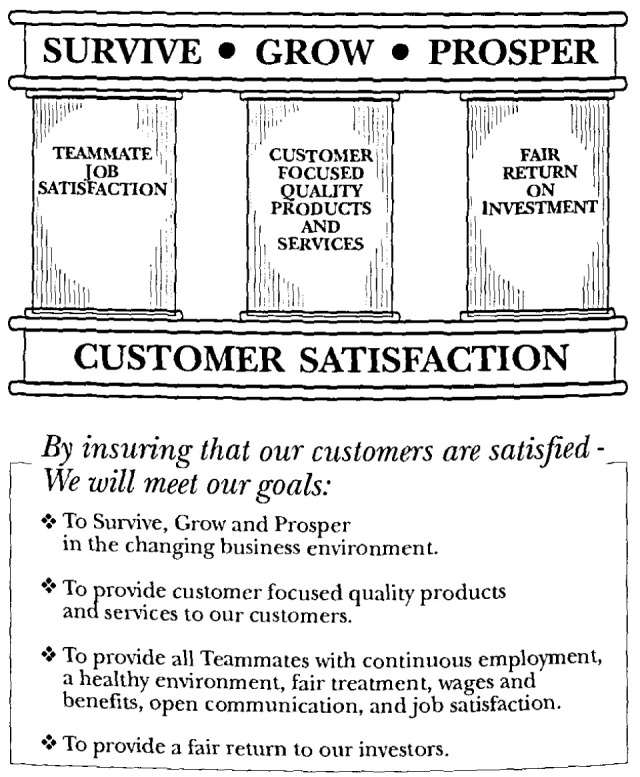
The Ames Team “Excellence Through Total Quality”.

Both companies, realizing that their survival hung in the balance, chose long-term solutions, not quick fixes. Ames inherited its quality princples and training from Xerox Corporation Busines Products and Systems, a 1989 Baldrige Award winner. Ames, a key supplier to Xerox, requested and received training for its Executive Committee in 1987. The smaller company quickly modified the Xerox “Leadership Through Quality” process to fit Ames’ size and culture (see Fig. 1). Marvil and his senior management devoted eight months to formulating strategy for the Ames Total Quality Process. When they were ready to apply their new process, the “Teammates” at Ames owned it, believed in it, and were determined to implement it.

Eastman, too, was prepared to commit time and financial and human resources to developing a Quality Policy and its Quality Management Process. CEO Deavenport understood that TQM could not be a pilot project of 6 months or even 6 years duration; it had to become a way of life. He provided a vision, and communicated it to his employees, along with the thought that as new measures were implemented to effect the new vision, employees had input. They could bring about thoughtful and continuous change. Empowered employees became more creative, energetic, enthusiastic, and productive, as management learned to, in Deavenport’s words, “stand back and let them do their jobs.”

Yet, with the strikingly positive results that both Ames and Eastman derived from their conversions to TQM, both emphasize that TQM is not *what* they do, but the *way* they do it. Both responded to customers, market demand, and, as the success of their new cultures of quality grew, so did their businesses.

## 3. Information and Analysis

Up-to-the-minute, accurate information and insightful analysis of that information makes a competitive-edge difference in today’s world marketplace. Benchmarking and competitive comparisons are ways in which businesses can set “stretch” goals for themselves to attain superior results.

Eastman considers itself “data-driven,” noting that information and analysis drive everything that follows—strategic planning, human resource management, operational results. Good information and analysis help Eastman to determine: i) whether the company’s processes are working, ii) if those processes are the best possible for Eastman, and iii) whether the processes are in concert with one another and contributing to success.

Access to information is also crucial in Eastman’s communications network of PCs, mid-range and mainframe computers, and distributed control systems. Within the network there are numerous information systems, two of which are vital for Eastman’s continued competitive stance. First, Manufacturing Information Systems are in each of the major manufacturing divisions. There, data are collected from distributed control systems, operators, and from laboratory test results, then used for statistical process control, scheduling, inventory, quality assurance, process evaluation, and process improvement.

The second major information system is GLOBIIS, or Global Business Integrated Information System. When totally in place, GLOBIIS will process information in 11 different languages and integrate all Eastman’s daily business functions into a single computer system.

Benchmarking has become routine at Eastman. Having learned early-on that performance indicators and goals have little use or credibility unless they are based on what the competition is doing, Eastman selected a core of 20 companies, 10 competitors and 10 customers, against which to compare performance data. All were chosen because of their market similarities to Eastman and for their track records of innovation and strategic and financial successes. Information gleaned from such benchmarking often has a direct, measurable effect. It was a benchmarking study that led to the company’s Rapid Globalization goal that significantly increased sales and assets outside the United States.

To keep a constant check on the relevance of all this information, Eastman organized its customers and markets into more than 40 distinct businesses called “units of analysis.” Based on the information coming from these units, Eastman makes significant business decisions: where to allocate capital, where to build relationships, and what kinds of services to provide. The company also does some 50 direct internal assessments yearly using the Malcolm Baldrige criteria. Results from these assessments help management determine whether the systems and processes are working in concert, and whether the company is achieving its vision, its goals.

At Ames, all information is collected and analyzed with the company goal and motto, “Survive, Grow and Prosper,” in mind. Five major types of data and information contribute to the company’s goal. *Customer-related data* are gathered in annual customer satisfaction surveys that measure performance in quality, cost, delivery, and service. Such survey data, when analyzed in a historical continuum, provide insight into the company’s competitive position. Similarly, Ames considers its suppliers as partners and uses *supplier-related data* to recognize superior performance or to spot a need for additional training.

*Produet and senke performance data* come from nine different sources, including simulated functional product testing, sales, checks for parts-per-million defects, and cost of quality. *Business process data*, including information on internal operations, facilitate Ames’ proactive approach to improving internal processe, providing training, and measuring Teammate satisfaction and involvement. The Execitive Committee evaluates operating costs and overhead against the company’s *cost and financial data.*

Ames began actively benchmarking in 1988. Since then, it has become a member of the International benchmark Clearinghouse and has provided training for selected Teammets. Ames benchmark in the areas of customers and suppliers, product and service quality, and internal operations, and is open to expanding its benchmarking efforts.

## 4. Strategic Quality Planning

Ames reviews and updates its Strategic Business Plan annually, fully integrating quality planning with goals to be achieved 5 years into the future. The plan, with a major section entitled “Total Quality,” in addition to traditional components such as “Sales and Marketing” and “Finance and Human Resources,” takes into account long- and short-term requirements of external and internal customers. Using the benefits of benchmarking even in planning, Ames adapted a concept from another Baldrige winner, The Wallace Company, to form seven Quality Strategic Objective teams that address various aspects of quality.

External customers’ expectations are defined by means of surveys, quality audits, direct interviews, purchase contracts and market studies, while internal customers’ needs generally include financial resources, workplace health and safety considerations, and Teammate recognition, empowerment, and training. A technology-oriented company, Ames also devotes a major section of the plan to research and development.

Eastman emphasizes that in its Strategic Quality Planning Process, “The planners are the doers.” Another over-arching principle is that planning must involve each dimension of Eastman’s organization: business organizations, functions, geographies, and core competencies.

The Strategic Quality Planning Process at Eastman is assessed annually to assure that the company plans and operates in a manner that provides greatest value for all stakeholders—suppliers, the public, investors, customers, and employees. Eastman divisions focus on key results areas for their organizational units and evaluate how performance in these areas contributes to overall company performance. At each level, teams own the planning process and are involved in and accountable for their plans and performances. They use a “Plan/Do/Check/Act” cycle for increased learning and standardizing to best practices. Eastman wants the company’s initiatives translated into meaningful expectations in everyone’s daily work. Management and employees have demonstrated a willingness to communicate, to be stakeholders and a passion about the Eastman mission.

## 5. Human Resource Development and Management

Ames considers its Teammates its scarcest and most valuable resource, and places high priority on programs to train and retain them. Ames’ high level of employee job satisfaction is due, in part, to a Labor Requirements Quality Improvement Plan, developed by a team of hourly and salaried employees. The company provides various opportunities for employee involvement and feedback: teammate involvement groups, quality improvement process and problem-solving process teams, a suggestion system, a direct line to the president, plant walk-arounds, social gatherings, cafeteria meetings, bulletin boards, and publications.

Employees are recruited based on Ames’ business plan. Each new Teammate receives 24 hours of basic “Excellence Through Total Quality” training and is assigned a coach. Understudy programs are available for technical professionals, as are programs in basic literacy. Ames also supports an off-site tuition assistance program for technical course work, undergraduate and graduate degree programs and personal development and enrichment.

Performance recognition ranges from a simple “Atta boy” to gifts of up to thousands of dollars. The quarterly company magazine, *The Echo*, lists at least two full pages in every issue of Teammates who have been honored. Press releases also are sent to local newspapers.

Employee health, safety, and security are foremost in company priorities. In addition to health and life insurance, retirement savings, and a pension plan, Ames provides influenza vaccines for employees and contracts with an industrial health physician who visits several times each year.

Eastman approaches all human resource objectives—empowerment, teamwork, diversity, well-being-through a structure of interlocking teams. The teams enable employees to develop goals and measures that are compatible and integrated with the company’s Major Improvement Opportunities. Eastman has found that such teams, interlocking up and down the supervisory chain, promote information sharing and feedback, innovation and ownership in managing work areas.

The company has removed traditional barriers to empowerment, modifying standard operating procedures, equalizing employee benefits, eliminating clock cards, and revising dress codes. Training needs are identified for each employee, placing emphasis on personal growth that is consistent with current job expectations and anticipated future requirements. Eastman’s performance assessment system removes employee labels and encourages involvement and ownership.

## 6. Management of Process Quality/Quality and Operational Results

For both Ames and Eastman, process quality and operational results begin with innovative product design and companywide communications networks taking into account the potential customer and market needs. At Eastman, multifunctional teams coordinate and integrate the sub-processes. The team members are given a Quality Management Facilitator, or coach, trained in statistical process control, and apply to their project an Eastman-developed quality implementation procedure called PECI, or Process Evaluation, Control, Improvement.

Process quality and operational results are assessed against the Baldrige criteria, the core of Eastman’s evaluative process over the past four years. In addition, the company uses ISO 9000 quality standards, and now has seven manufacturing units registered to ISO 9000.

Eastman has shown three dramatic and measurable results after having applied the tools of quality to their manufacturing operations. The first is superior return on assets; compared to the companies against which Eastman benchmarks, Eastman has had the best profit margin since 1990. The next result, aggressive sales revenue growth, is particularly impressive; Eastman’s annual sales have grown 40 percent since 1988. Last, Eastman has actively sought rapid globalization and succeeded, doubling sales outside the United States since 1984.

Eastman credits use of total quality management for much of its success. The company is obviously proud of its involved, committed, continually improving employees, but is also quick to say its quality journey is just that—a journey—not a destination.

Ames translates its strong commitment of leadership and teamwork into management of process quality for several hundred different products being manufactured in numerous ways. It sees the key element in a successful customer relationship as an exchange of information through continuous customer and supplier involvement, from prototype design to pilot plant to full-scale production and delivery. Throughout the process, built-in controls-systems checks, supplier audits, visual inspections and laboratory testing—ensure that Ames and the customer are satisfied with the product. Avoiding problems is the goal. “Prevention,” Ames says, “not correction, is our policy.”

The caution pays off. Ames has increased price reductions to its customers from $200,000 in 1990, to $5,300,000 in 1993. Its on-time delivery improved from 81 percent in 1989, to 98.6 percent in 1993. Corporate quality, as measured by defective parts per million, improved exponentially; Ames reduced its defective parts from 33,841 per million in 1989 to 11 in 1993. At the same time, as a planned consequence of the conscious commitment to quality process, operational quality, efficiency and workplace safety improved.

## 6. Customer Focus and Satisfaction

Based on the criteria which includes product quality and uniformity, supplier integrity, correct delivery, and reliability, over 70 percent of Eastman’s customers rate the company “Number One.” Eastman’s high customer ratings reflect the company’s ability to anticipate, understand, and meet customer needs.

**Fig. 2:**
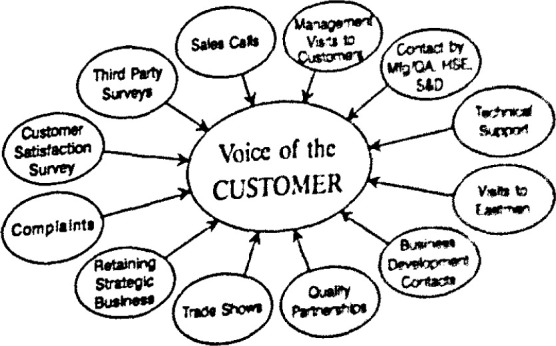
Methods for Collecting Eastman customer information.

Eastman forges strong relationships with customers through technically trained field sales and technical service representatives, sales support specialists, customer service specialists, and others. The contact frequently is made face-to-face. Also, a toll-free number for sales or service is available to customers and potential customers 7 days a week, 24 hours a day. A Strategic Intent Vision (Making Eastman the “World’s Preferred Chemical Company”) drives the organization to go beyond meeting customer needs to exceeding them. In contrast to the practice of many manufacturers, Eastman’s sales force is not the only customer contact, but the first of many. Management, manfacturing units, quality assurance, health, safety and environment and supply and distribution departments are also in contact with customers in person or by phone (see Fig. 2).

The company provides customer-contact employees with important, tangible tools to maintain and improve Eastman’s enviably high level of customer satisfaction. In addition to electronic data interchange with customers and advance notice of product changes, Eastman offers a simplified warranty on its Conditions of Sale and a no-fault return policy for plastics that is believed to be unique in the plastics industry.

Ames focuses on certain basic elements of its customers’ perceptions of value: total quality of products and services, Ames’ position as a technology leader, price, attention to customers, and ease of doing business. An annual survey, conducted by an outside, independent firm, asks customers to rate Ames in terms of total quality of such basic elements. Two additional surveys, a quarterly customer satisfaction survey and a monthly customer contact survey, both conducted by the sales department, give Ames an idea of how its doing in customers’ eyes.

A customer service group collaborates with manufacturing management to set service standards through benchmarking and to incorporate changes into the Ames customer service manual. The company’s performance in increasing its customer focus and satisfaction is reviewed monthly. As measured by a 60 percent decrease in occurrences of customer dissatisfaction, the system is effective, yet Ames states that it will constantly seek ways to improve.

## 8. Public Responsibility and Corporate Citizenship

Ames Rubber Corporation, located in rural Sussex County, New Jersey, is proud and protective of its home. Ames considers the surrounding communities its customers, and places its responsibility to the public’s health, safety, and the environment first. Long before New Jersey became one of the most stringently environmentally regulated states Ames formed a Department of Regulatory Affairs to address issues of waste disposal, water contamination, air emissions, and solvent usage. Even so, a company analysis showed that historically accepted practices could lead to substantial liability; thus, Ames adopted a proactive approach, preferring prevention to control, and spent over two million dollars in site evaluations and clean-up in 1992 alone.

Progress is evident in many processes. Ames has eliminated solvent-stripping processes, will soon implement solventless degreasing, and is testing aqueous-base adhesive systems. The company controls emissions from its manufacturing sites by thermal oxidation, has constructed a modern hazardous waste storage pad, and developed a program to recycle spent activated carbon from water.

Ames puts equal emphasis on protecting its Teammates. The company’s safety record stands as almost twice as good as the industry average, and is a benchmark for Ames’ insurance carriers.

Ames management encourages corporate and employee citizenship. Teammates donate time to many community outreach activities: local fire and rescue squads, Big Brothers, Big Sisters, United Way of Sussex County, March of Dimes, local athletic programs and holiday food drives. The company policy is that good corporate citizenship goes far beyond providing employment and paying taxes. As well as contributing financial resources to worthy causes in the community, Ames shares the total quality concept with other companies and with nearby colleges and universities, including Rochester Institute of Technology, New Jersey Institute of Technology, and Lehigh University.

Eastman is a highly visible participant in Responsible Care^®^, an initiative undertaken by the Chemical Manufacturers Association, with Eastman as a key contributor, to protect the environment and to meet or exceed the relevant regulations at local, state, and national levels. Eastman is recognized as an industry model for phasing out chlorofluorocarbons as coolants in refrigeration and air conditioning systems. The company also maintains its own wastewater and other waste treatment facilities.

Eastman strives to be a good neighbor, promoting a free flow of environmental information to the surrounding communities, opening its plants for tours and open houses, inviting interviews from the media, publishing newsletters, and sponsoring a special environmental hot line. The company takes an active lead in recycling plastics, forming a partnership with a waste management firm to provide recycling services in four southern states. The project was cited as best in the United States in 1991 by “Keep America Beautiful.”

As with many organizations committed to the principles of quality, Eastman and its employees are active in community programs: Junior Achievement, recycling fairs, chambers of commerce, and other service organizations. In monetary contributions alone, Eastman gives over 2 million dollars each year to charitable and educational organizations. In addition, work schedules can be arranged, so that employees can speak to school classes, deliver “Meals on Wheels” or spend time on the “Putting Children First” project, where employees in the Kingsport area and local educators have a partnership and long-term commitment to increase students’ competence in math, science, and technology. Similarly, Eastman’s Texas plant has entered into a business and education coalition called GLOBE, or Greater Longview Organization for Business in Education. More than 25,000 students have benefitted from this nationally recognized model of partnership in business and education.

## 8. Quality in Research and Development

With efforts in basic and applied research, including pilot plant operations, Eastman was one of the first to ask whether TQM could really work—especially in research. Beginning in 1987, Eastman included researchers on interlocking teams to identify processes and customers and to design experiments. However, after taking stock of their efforts in late 1989, Eastman saw that there was no significant improvement with its research initiatives. In fact, there was considerable griping about time spent in meetings and wasted money and effort.

In the final analysis, Eastman realized that the focus should have been on the main output of research: new and improved product and process concepts that were desirable in the marketplace. In 1990, the company instituted an annual fact-finding analysis system to evaluate opportunities for improvement. In 1991, it placed more emphasis on concept development teams. In 1992, new research liaison teams ensured better linkage with in-house business organizations, and in 1993, the research-customer interaction process was further refined. At each of these stages, Eastman realized an improvement in efficiency and an increase in output, while resource and people levels remained constant. Eastman emphasizes that it has changed its system, not the scientists, and the difference is measured in tens of millions of dollars per year savings to the company. The scientists themselves agree; in 1990, only 15 percent felt that they were contributing innovatively to research productivity. By 1993, after the shift in philosophy to search concepts, 90 percent thought of themselves working to improve research productivity.

Like Eastman, Ames was sensitive to a common concern that TQM processes might inhibit the creative spirit inherent in research. Ames recognized that in its customer-driven world, the internal customers involved in research and development should receive just as much consideration as the external customers. Teammates in R&D interact regularly with their contacts in sales and marketing, the manufacturing divisions for factory service and for new products, and with the external customers.

Compared with like-sized manufacturers, Ames returns a high percentage of revenue to its R&D budget. Because the company sells in a very competitive market, it is committed to “cutting edge” materials and processes. By their measure of R&D performance, (taking into account material use, labor use, process capability, yield, total product cost, and cycle time), Ames is confident that it has fully integrated total quality management into its research and development process.

## 9. Conference Summary

Dr. Joseph M. Juran’s reference literature, training courses, books, and video cassettes on quality management have been translated into 16 languages. His work has been formally honored by 12 countries with over 40 national, international, and foreign awards, including the Order of the Sacred Treasure, awarded by the Emperor of Japan and the National Medal of Technology, presented by, the President of the United States. The following is a summary of highlights from his comments at the close of Quest for Excellence VI and h,s prognosis for quality in the future.

The two companies portrayed in depth both described wake-up calls as the impetus that started them on their quality journeys. As Eastman and Ames bo learned, leadership has certain non-delegable roles; setting quality goals; personally presiding over a quality council; providing a vision statement, training and resources; establishing new performance goal; and being present for employee recognition. “No company has reached world-class quality status,” Juran says, “without the personal involvement of senior management.” Juran commended Ames and Eastman for getting “past the fog of buzzwords into the terrain of reality,” defining values and establishing benchmarks.

Of the information and analysis systems developed by both companies. Juran observed that Ames and eastman were provided with a broad array of measurment of quality of business, akin to the measurment means taken for granted by the technological world for centuries. In the 1980s, may U.S. companies had had no early warning of the foreign invasion. Juran says that now, through quality managements, companies have useful market indicators.

Human relations, in Juran’s estimation, have undergone irreversible change. At the beginning of the century, management was essentially by fiat, an approach that was in part responsible for U.S. ascendance to the height of industrial power. Although circumstances could be harsh and workers were largely unempowered, such a management style was warranted when educational levels and the literacy rate in English were low. Today, such a management philosophy is obsolete, and the consequence of that obsolescence is an underemployed workforce. The quality focus on involvement, training, accountability, communication, and empowerment has been, Juran says, “a watershed event” in human relations. Juran says of business, that it is a collection and series of processes, interdependent and synergistic, much like the parts and processes of the human body.

“Both companies assessed themselves against the Baldrige Award criteria and found no better standard,” Juran observed. Noting that the Office of Quality Programs at the National Institute of Standards and Technology mails thousands of packets of information yearly, yet only around 100 companies apply, Juran is convinced that many other companies assess themselves privately against the Baldrige criteria and make changes.

“Where is the United States?” Juran asks. “In 1990,” he recalls, “I declared that we had a break in the clouds; it was okay to be optimistic.” He sees continued progress from his present 1994 perspective. He is not so optimistic for Europe, though, citing the continent’s preoccupation with the ISO 9000 series. Juran allows that ISO 9000 has pluses as a documentation system, but it lacks emphasis on the need for senior leadership, training, empowerment, and continued improvement

“Japan continues to improve, but,” predicts Juran, as the United States closes the quality gap the force of patriotism takes over.” “Now “ he says’ “the gap is not so large that it’s undismissable “ He concedes that perceptions are usually longer lasting than facts, but as perceptions change, customers will return to American goods. Juran says that if the twentieth century was marked by U.S. leader ship in productivity, in the twenty-first century, quality will be the major area of world competition

Dr. Curt Reimann, Director of the Office of Quality Programs at the National Institute of Standards and Technology, closed the Conference encouraging participants to take to heart, and to take home, the examples of community involve ment and dedication set by Ames and Eastman on their quality journeys.

## 11. General References

1994 Criteria for the Malcolm Baldrige National Quality Award.

Conference notebook, Quest for Excellence VI, February 7-9, 1994, Washington, DC.

